# Accumulative Effects of Multifrequency Microwave Exposure with 1.5 GHz and 2.8 GHz on the Structures and Functions of the Immune System

**DOI:** 10.3390/ijerph20064988

**Published:** 2023-03-12

**Authors:** Chuanfu Yao, Ji Dong, Ke Ren, Liu Sun, Hui Wang, Jing Zhang, Haoyu Wang, Xinping Xu, Binwei Yao, Hongmei Zhou, Li Zhao, Ruiyun Peng

**Affiliations:** Beijing Institute of Radiation Medicine, Beijing 100850, China

**Keywords:** microwave, multi-frequency, immune system, effect

## Abstract

Microwave ablation can produce immune activation due to thermal effects. However, the nonthermal effects of microwaves on the immune system are still largely unexplored. In this study, we sequentially exposed rats to 1.5 GHz microwave for 6 min and 2.8 GHz microwave for 6 min at an average power density of 5, 10, and 30 mW/cm^2^. The structure of the thymus, spleen, and mesenteric lymph node were observed, and we showed that multifrequency microwave exposure caused tissue injuries, such as congestion and nuclear fragmentation in lymphocytes. Ultrastructural injuries, including mitochondrial swelling, mitochondrial cristae rupture, and mitochondrial cavitation, were observed, especially in the 30 mW/cm^2^ microwave-exposed group. Generally, multifrequency microwaves decreased white blood cells, as well as lymphocytes, monocytes, and neutrophils, in peripheral blood, from 7 d to 28 d after exposure. Microwaves with an average density of 30 mW/cm^2^ produced much more significant inhibitory effects on immune cells. Moreover, multifrequency microwaves at 10 and 30 mW/cm^2^, but not 5 mW/cm^2^, reduced the serum levels of several cytokines, such as interleukin-1 alpha (IL-1α), IL-1β, interferon γ (IFN-γ) and tumor necrosis factor α (TNF-α), at 7 d and 14 d after exposure. We also found similar alterations in immunoglobulins (Igs), IgG, and IgM in serum. However, no obvious changes in complement proteins were detected. In conclusion, multifrequency microwave exposure of 1.5 GHz and 2.8 GHz caused both structural injuries of immune tissues and functional impairment in immune cells. Therefore, it will be necessary to develop an effective strategy to protect people from multifrequency microwave-induced immune suppression.

## 1. Introduction

In recent years, microwave technologies have been rapidly developed and widely applied in various fields, such as medicine, navigation and communication systems, and high-voltage transmission and transformation systems [1,2]. The central nervous system (CNS), cardiovascular system, reproductive system, and hematopoietic system are sensitive biological organs to microwave radiation. However, the interaction of microwaves and biological systems is still largely unexplored [3,4]. Studies suggested that microwaves can act as a helping agent to produce genetic alterations in biosystems. In detail, microwaves can induce indirect thermal effects and direct nonthermal effects on biomacromolecules and bulky molecular units, which contribute to physiological alterations [5]. Resonance is an important mechanism for microwave-biological system interaction. Resonance causes electrons or ions to vibrate, destroying the structures of biomacromolecules, such as proteins, and impairing the activity of proteins [6,7]. Microwave-induced increase of reactive oxygen species (ROS) is another pivotal mechanism for microwave-biological system interaction [8]. Studies have demonstrated that microwave-induced biological effects are associated with the frequency, power density, and duration of exposure [9,10,11]. Due to the complex environment of the electromagnetic field in our daily life, people are always exposed to microwaves with different parameters. For example, L band microwaves ranging from 1.0 GHz to 2.0 GHz and S band microwaves ranging from 2.0 GHz to 4.0 GHz could be simultaneously emitted by communication weather satellites and synthetic aperture radar [12,13]. However, most studies on the biological effects of microwaves have focused on single-frequency microwaves. Our group previously reported the accumulative effects of microwaves with different frequencies on cognitive function and the tissue structure in the nervous system [14,15].

The immune system, composed of the innate immune system and adaptive immune system, plays critical roles in defense against infection and cellular mutation. Microwave ablation, which can enhance the therapeutic responses of immune checkpoint inhibitors and cell immunotherapy, has emerged as an effective adjuvant therapy for cancer [16,17,18]. Most of the studies focused on microwave-induced thermal effects, which in turn activated the immune system by inducing the T-helper 1 cell (Th1) immune response and activating natural killer cells [19,20]. A few studies have reported the nonthermal effects of microwaves on the immune system. However, the results are still controversial.

In this study, we found that multifrequency microwaves with a frequency of 1.5 GHz and 2.8 GHz dose-dependently caused histopathological injuries in the thymus, spleen, and mesenteric lymph nodes. Multifrequency microwaves also decreased the levels of major types of immune cells. Microwaves powers of 10 and 30 mW/cm^2^, but not 5 mW/cm^2^, reduced the concentration of both cytokines and immunoglobulins (Igs) in peripheral blood. This study will provide a basis for the prevention and treatment of immune suppression induced by multifrequency microwave radiation.

## 2. Materials and Methods

### 2.1. Animals and Microwave Exposure

One hundred eight 6- to 8-week-old male Wistar rats (180 g ± 20 g) (Charles River, China) were randomly divided into four groups: the sham-exposed (Sham) group and the 5 mW/cm^2^ (LS5), 10 mW/cm^2^ (LS10) and 30 mW/cm^2^ (LS30) multifrequency microwave-exposed groups (n = 27 per group). The animal experiments were approved by the Institutional of Animal Care and Use Committee at the Beijing Institute of Radiation Medicine (Beijing, China), and all experimental procedures were performed in accordance with the National Institutes of the Health Guide for the Care and Use of Laboratory Animals.

The microwave exposure system, based on a klystron amplifier model JD 2000 (Vacuum Electronics Research Institute, Beijing, China), had been described previously [21]. The microwave source could generate pulsed microwaves at the L band with a frequency of 1.5 GHz and the S band with a frequency of 2.8 GHz. The L band microwave pulses were delivered at 250 pps, 500 pps, and 1500 pps, respectively, with a pulse width of 200 ns. The S band microwave pulses were delivered at 50 pps, 100 pps, and 300 pps, respectively, with a pulse width of 500 ns. Microwave energy was transmitted via a rectangular waveguide and A16-dB standard-gain horn antenna to an electromagnetic shield chamber (7 × 6.5 × 4 m). The diagonal of the antenna was 33 cm. The interior walls of the chamber were covered with 500 mm and 300 mm pyramidal microwave absorbers to minimize reflections (>45 dB). The emitted power was measured with a semiconductor detector connected to a directional coupler at one circulator port and displayed on an oscilloscope. The distance from the antenna to the top of the animal cage was 98 cm. The schematic of the experimental and exposure setup is shown in Supplementary Figure S1.

Rats in the LS5, LS10, and LS30 groups were sequentially irradiated by 1.5 GHz and 2.8 GHz microwaves for one cycle. Briefly, rats were exposed to 1.5 GHz microwave for 6 min, followed by 2.8 GHz microwave for another 6 min at average power densities of 5, 10, and 30 mW/cm^2^, respectively. The power densities were measured with a calibrated waveguide antenna as described previously [15]. Rats in the sham group were processed in parallel with those in the microwave exposure groups but without microwave exposure.

### 2.2. Histopathological Analysis

At 6 h, 7 d, 14 d, and 28 d after microwave exposure, rats in each group were euthanized, and the thymus, spleen, and mesenteric lymph nodes were removed. After fixation in a 10% buffered formalin solution (Sinopharm, Beijing, China), the tissues were embedded in paraffin and cut at a thickness of 3 μm in the coronal plane. The sections were stained with hematoxylin and eosin (H&E) (Leagene Biotechnology, Nanjing, China) according to the manufacturer’s instructions. The histopathological alterations in corresponding tissues were observed by light microscopy (Leica, Germany).

### 2.3. Transmission Electron Microscopy (TEM)

The thymus and spleen of rats were collected at 7 d after exposure, as described above. The tissue blocks were fixed in 2.5% glutaraldehyde (Merck, Rahway, NJ, USA) and 1% osmium acid (Sinopharm, China) in sequence, processed with graded ethyl alcohols (Sinopharm, China), embedded in EPON618 with 70-nm thin slices, laid on copper mesh and then stained with heavy metals, uranyl acetate (Henye Zhongyuan Chemical, Beijing, China), and lead citrate (Sinopharm, China). The ultrastructures of the thymus and spleen in the indicated groups at 7 d after exposure were observed and photographed by TEM (Hitachi, Tokyo, Japan).

### 2.4. Analysis of Immune Cells in Peripheral Blood

At 6 h, 7 d, 14 d, and 28 d after microwave exposure, rats in the indicated groups were anesthetized by 1% pentobarbital sodium (Foshan Chemical, Foshan, China) at a concentration of 30 mg/kg. Then, heparin anticoagulant peripheral blood was collected from the inferior vena cava (IVC). The types of immune cells in peripheral blood, such as white blood cells, lymphocytes, and neutrophils, were analyzed by an automatic blood cell counter (Sysmex Europe GmbH, Hamburg, Germany).

Moreover, the immunophenotypes of lymphocytes, including T lymphocytes and B lymphocytes, were detected by BD Accuri C6 flow cytometry (BD, Franklin Lakes, NJ, USA). The antibodies were as follows: CD45, CD3, and CD45R (eBioscience, San Diego, CA, USA).

### 2.5. Cytokine Release

At 6 h, 7 d, and 14 d after multifrequency microwave exposure, peripheral blood was collected, and then the sera were separated by centrifugation. The levels of various cytokines, including IL-1α, IL-1β, IL-4, IL-6, IL-10, IL-12p70, tumor necrosis factor α (TNF-α), and interferon γ (IFN-γ), were analyzed by the Cytokine & Chemokine 22-Plex Rat ProcartaPlex Panel (Invitrogen, San Francisco, CA, USA) using the Luminex 100 system (Luminex, Austin, TX, USA).

### 2.6. Concentration of Immunoglobulins and Complement Proteins in Sera

At 6 h, 7 d, and 14 d after microwave exposure, serums were collected from peripheral blood. The levels of immunoglobulins (Igs) in serum, including IgA IgM and IgG, were analyzed by enzyme-linked immunosorbent assay (ELISA) (MultiSciences, Hangzhou, China) after diluted by 1:1000, 1:10,000, and 1:100,000. Moreover, the levels of complement proteins in serum, namely, complement 3 (C3) and C4, were also detected by ELISA (CUSABIO, Wuhan, China) after dilution of 1:200 and 1:1000, respectively.

### 2.7. Statistical Analysis

Data are presented as the mean ± s.e.m. and analyzed by GraphPad Prism software version 6.0 (GraphPad Software, San Diego, CA, USA). Longitudinal data were analyzed by two-way repeated-measure ANOVA followed by Bonferroni post hoc tests. Differences were considered significant at two-sided *p* < 0.05.

## 3. Results

### 3.1. Multifrequency Microwave Dose-Dependently Caused Structural and Ultrastructural Injuries

**Figure 1:**
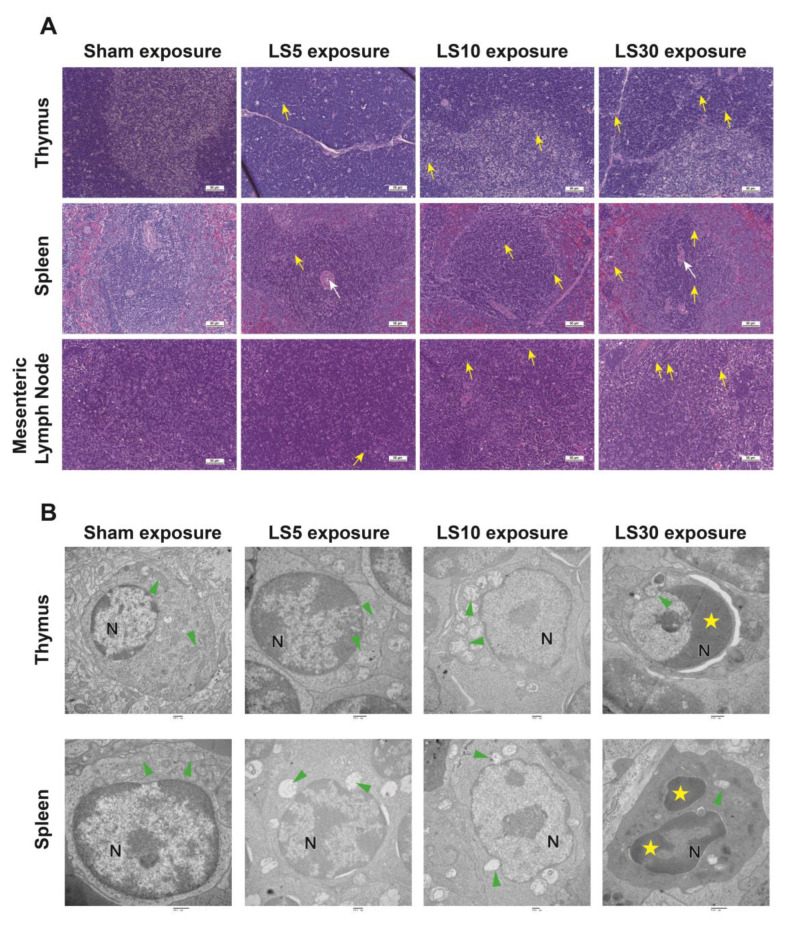
Structural injuries of the thymus, spleen, and mesenteric lymph nodes at 7 d after exposure to multifrequency microwaves. Male Wistar rats were sequentially exposed to 1.5 GHz and 2.8 GHz microwaves at average power densities of 5 (LS5), 10 (LS10), and 30 (LS30) mW/cm^2^ as described in the Materials and Methods. Representative photomicrographs of the thymus, spleen, and mesenteric lymph nodes (**A**) and representative electron micrographs of the thymus and spleen (**B**) at 7 d after exposure. The yellow arrows indicate lymphocyte nuclear fragmentation, and the white arrows indicate tissue sinus congestion in Figure 1A (Scale bar = 50 μm). The letter N represents the nucleus of the cell, the green triangle indicates the mitochondria in the cell, and the yellow pentagon indicates the nucleus fragment in Figure 1B (Scale bar = 500 nm).

The histopathological alterations in the thymus, spleen, and mesenteric lymph nodes were evaluated in rats exposed to multifrequency microwaves of 1.5 GHz and 2.8 GHz at the indicated average power densities for 6 min. H&E staining showed distinguishable cortex and medulla and highly proliferative lymphocytes in the thymus in the Sham group. Vascular dilatation and congestion, and nuclear fragmentation of lymphocytes could be detected in the thymus at 7 d and 14 d after exposure to multifrequency microwaves at an average power density of 5 mW/cm^2^, 10 mW/cm^2^ and 30 mW/cm^2^ and then recovered at 28 d after exposure. In the spleen, vascular dilatation and congestion were observed in all microwave-exposed groups, while nuclear fragmentation of lymphocytes was occasionally detected in the 10 mW/cm^2^ and 30 mW/cm^2^ microwave-exposed groups at 7 d and 14 d after multifrequency microwave exposure. Moreover, similar results were also observed in mesenteric lymph nodes (Figure 1A). The histopathological structures of the thymus, spleen, and mesenteric lymph nodes gradually recovered to normal at 28 d after exposure.

Furthermore, the ultrastructural injuries of the thymus and spleen were also analyzed by TEM at 7 d after exposure. TEM showed intact cellular ultrastructure, well-arranged chromatin in the nucleus, and transversely and longitudinally distributed mitochondria in both the thymus and spleen in the Sham group. Both 5 mW/cm^2^ and 10 mW/cm^2^ multifrequency microwaves caused mitochondrial swelling and mitochondrial cristae rupture, and 30 mW/cm^2^ multifrequency microwaves resulted in agglutination of nuclear protein, perinuclear space widening, and cytoplasmic pyknosis in lymphocytes of the thymus. In the spleen, 5 mW/cm^2^ multifrequency microwave exposure induced mitochondrial swelling and mitochondrial cristae rupture, while 10 mW/cm^2^ multifrequency microwave exposure caused mitochondrial swelling and mitochondrial cavitation in lymphocytes. Moreover, chromatin condensation, fragmentation, and cytoplasmic pyknosis were detected in the 30 mW/cm^2^ multifrequency microwave-exposed group (Figure 1B). The data suggested that multifrequency microwaves caused histopathological injuries in the thymus, spleen, and mesenteric lymph nodes, which was positively associated with the average power density.

### 3.2. Multifrequency Microwave Exposure Decreased Immune Cells in Peripheral Blood

**Figure 2:**
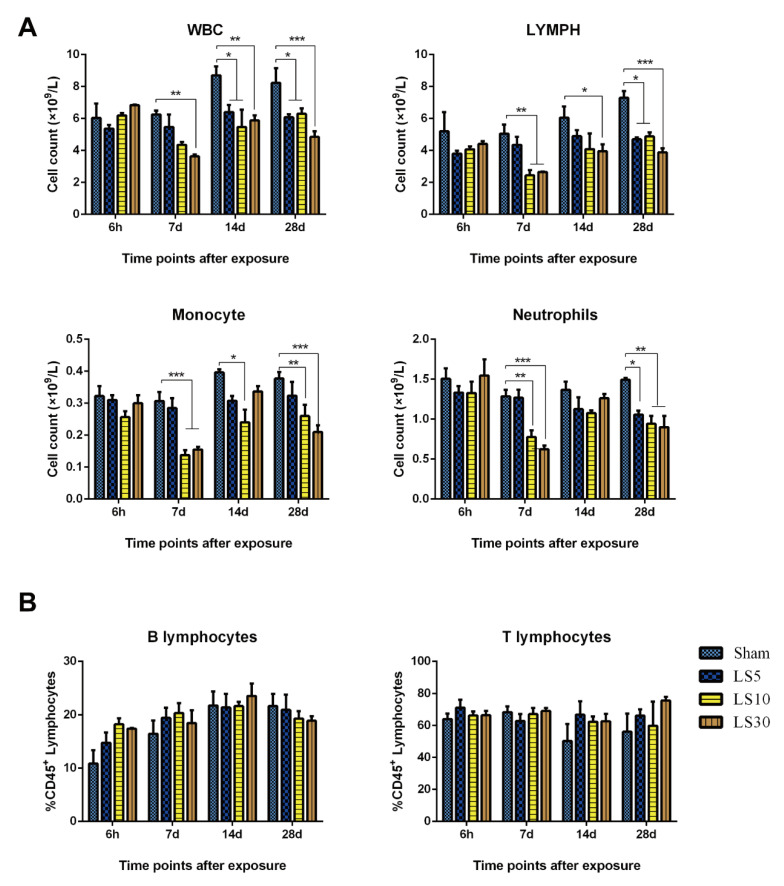
Multifrequency microwaves reduced white blood cells, including lymphocytes, monocytes, and neutrophils in peripheral blood. Wistar rats were exposed to multifrequency microwaves at average power densities of 5, 10, and 30 mW/cm^2^. At 6 h, 7 d, 14 d, and 28 d after exposure, heparin anticoagulant peripheral blood was collected from the inferior vena cava (IVC). The number of white blood cells, lymphocytes, monocytes, and neutrophils was analyzed by an automatic blood cell counter (**A**). Moreover, the immunophenotypes of lymphocytes, including T lymphocytes and B lymphocytes, were detected by flow cytometry (**B**). Data are shown as the mean ± s.e.m., * *p* < 0.05, ** *p* < 0.01, *** *p* < 0.001, vs. the corresponding groups.

White blood cells, mainly composed of lymphocytes, granulocytes, and monocytes, are pivotal effectors of the body’s defense system and can be rapidly activated by both internal and external stimuli. Here, we found that multifrequency microwaves significantly reduced the number of white blood cells from 7 d to 28 d after exposure, while 30 mW/cm^2^ microwaves produced much stronger suppressive effects. Moreover, the subtypes of white blood cells, including lymphocytes, monocytes, and neutrophils, were also decreased by multifrequency microwave from 7 d to 28 d after exposure, while no obvious changes could be detected at 6 h after exposure (Figure 2A). Furthermore, the inhibitory responses of lymphocytes, monocytes, and neutrophils in the 10 and 30 mW/cm^2^ groups were much stronger than those in the 5 mW/cm^2^ group. The results suggested that multifrequency microwaves produced suppressive effects on immune cells in peripheral blood, and the inhibitory responses were enhanced with increasing average power density. We further analyzed the phenotypes of lymphocytes, and the data suggested that the percentage of B lymphocytes was slightly increased at 6 h after exposure. However, no obvious statistical difference could be detected (Figure 2B).

### 3.3. Multifrequency Microwave Exposure Reduced Th1 Cytokines in Peripheral Blood

**Figure 3:**
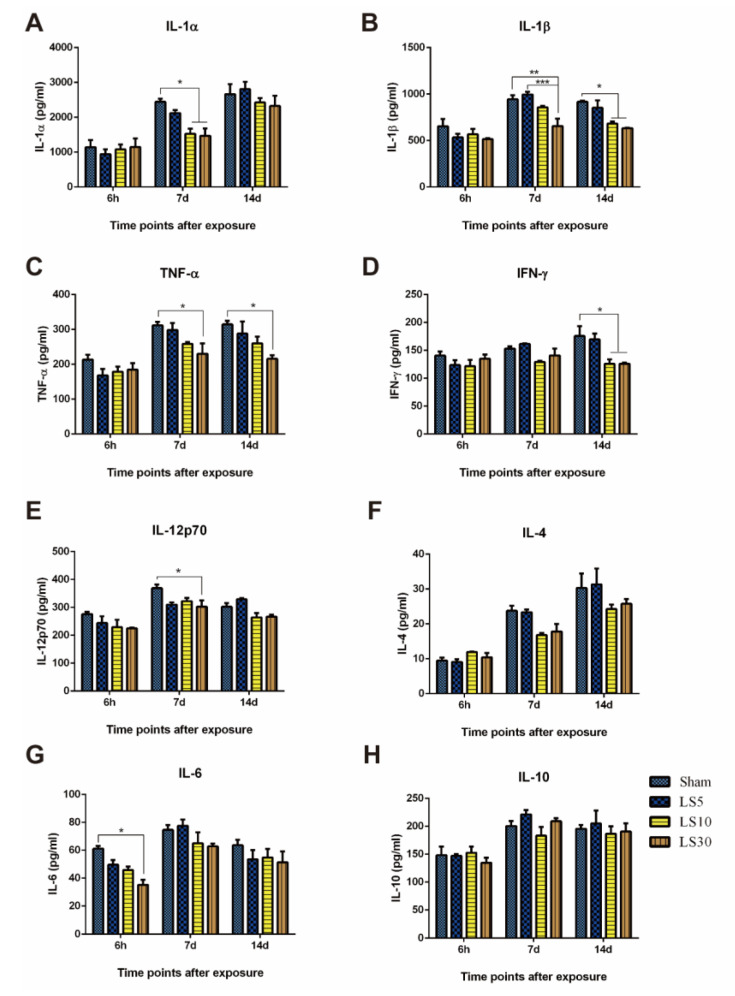
Multifrequency microwave exposure decreased the levels of Th1 cytokines in peripheral blood. Wistar rats were exposed to multifrequency microwaves at average power densities of 5, 10, and 30 mW/cm^2^. At 6 h, 7 d, and 14 d after exposure, peripheral blood was collected, and serum was separated by centrifugation. The levels of interleukin (IL)-1α (**A**), IL-1β (**B**), tumor necrosis factor α (TNF-α) (**C**), interferon γ (IFN-γ) (**D**), IL-12p70 (**E**), IL-4 (**F**), IL-6 (**G**), and IL-10 (**H**) were analyzed by Cytokine & Chemokine 22-Plex Rat ProcartaPlex Panel using flow cytometry. Data are shown as the mean ± s.e.m., * *p* < 0.05, ** *p* < 0.01, *** *p* < 0.001, vs. corresponding groups.

Cytokine expression and release have emerged as critical mechanisms by which immune cells respond to internal and external stimuli. In addition to altering immune cell numbers, the cytokine levels in peripheral blood, indicators of immune functions, were also analyzed at 6 h, 7 d, and 14 d after multifrequency microwave exposure. IL-1α (Figure 3A) and IL-1β (Figure 3B), which are closely related to the activation of T lymphocytes and maturation of B lymphocytes, were reduced in the 10 and 30 mW/cm^2^ microwave-exposed groups but not in the 5 mW/cm^2^ microwave-exposed group. Moreover, 30mW/cm^2^ multifrequency microwaves decreased TNF-α (Figure 3C) secretion both at 7 d and 14 d after exposure and reduced release of IFN-γ (Figure 3D) at 14 d and IL-12p70 (Figure 3E) at 7 d after exposure, which resulted in impaired functions of T lymphocytes. However, no obvious alterations in Th2 cytokine levels, such as IL-4 (Figure 3F), IL-6 (Figure 3G), and IL-10 (Figure 3H), were detected in peripheral blood, and only a transient decrease in IL-6 could be observed at 6 h after exposure. In addition, because of the contribution of Th2 cytokines to the activation of B lymphocytes, we speculated that multifrequency microwaves produced much stronger suppressive responses on T lymphocytes than on B lymphocytes.

### 3.4. Multifrequency Microwave Exposure Dose-Dependently Reduced the Levels of IgG and IgM in Peripheral Blood

**Figure 4:**
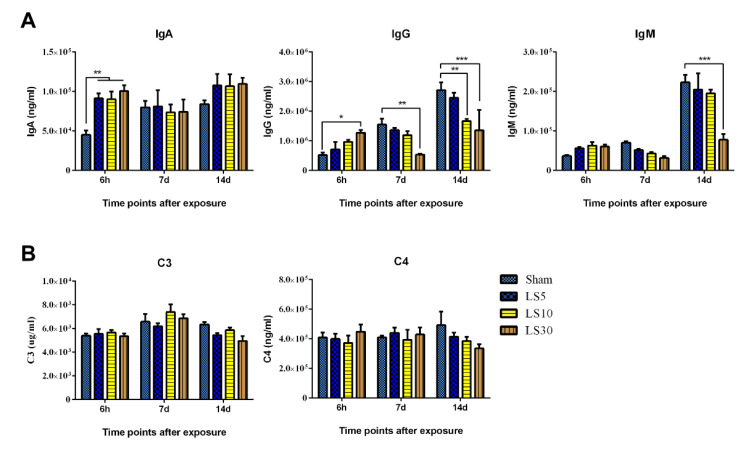
Multifrequency microwaves decreased the levels of IgG and IgM but did not complement proteins in peripheral blood. Wistar rats were exposed to multifrequency microwaves at average power densities of 5, 10, and 30 mW/cm^2^. At 6 h, 7 d, and 14 d after exposure, peripheral blood was collected, and serum was separated by centrifugation. The levels of immunoglobulins (IgA, IgM, and IgG) (**A**) and complement proteins (C3 and C4) (**B**) were analyzed by enzyme-linked immunosorbent assay (ELISA). Data are shown as the mean ± s.e.m., * *p* < 0.05, ** *p* < 0.01, *** *p* < 0.001, vs. corresponding groups.

Antibody-mediated immunity, such as antibody-mediated neutralization and antibody-dependent cell cytotoxicity (ADCC) effects, is a major mechanism of the humoral immunity process. Three pivotal antibodies, namely, IgA, IgG, and IgM, were analyzed at 6 h, 7 d, and 14 d after exposure to multifrequency microwaves (Figure 4A). The concentration of IgA was transiently increased in microwave-exposed groups at 6 h after exposure, while there was no obvious difference between the microwave-exposed groups and the sham group at 7 d and 14 d after exposure. For IgG, 30 mW/cm^2^ multifrequency microwave increased IgG levels at 6 h after exposure but reduced IgG levels at 7 d and 14 d after exposure. For IgM levels, a significant decline could be observed at 14 d after exposure to 30 mW/cm^2^ multifrequency microwaves. The transient stimulation of the secretion of antibodies at 6 h after exposure might be attributed to a compensatory mechanism, while the decrease in B lymphocytes might contribute to the decrease in antibodies at 7 d and 14 d after exposure.

Complement proteins, which play important roles in humoral immunity, can be activated by antibody-dependent and antibody-independent pathways. C3 and C4 are two major components in the complement system. In this study, we analyzed the levels of C3 and C4 in the peripheral. We found that multifrequency microwave exposure could slightly decrease C3 and C4 levels at 14 d after exposure (Figure 4B). However, no significant difference could be detected. Our data suggested that C3 and C4 were not affected by multifrequency microwave exposure.

## 4. Discussion and Conclusions

In recent decades, concern about the potential health hazards induced by microwaves has been rapidly growing [22,23]. Moreover, the International Agency for Research on Cancer (IARC) has classified electromagnetic waves as “possibly carcinogenic to humans” (Group 2B) [24]. It has been demonstrated that microwaves cause obvious injuries in various tissues and organs, such as the nervous system, reproductive system, and cardiovascular system [1,25,26]. Most reports evaluated the biological effects and corresponding mechanisms using microwaves with a single frequency at the indicated average power density [27,28,29]. Our group reported that different frequency microwaves, including the S band, L band, and X band, impaired hippocampal functions, caused cardiac injury, and damaged the blood-testis barrier via multiple mechanisms [10,30,31,32]. However, people are always exposed to such a complex environment in daily life, which includes microwaves with varied frequencies, power densities, and so on. The L (1.0 GHz to 2.0 GHz), S (2.0 GHz to 4.0 GHz), and C (4.0 GHz to 8.0 GHz) band microwaves are widely used in daily life, such as in medicine and communication. Our group previously reported the accumulative effects of 1.5 GHz and 2.8 GHz, as well as 1.5 GHz and 4.3 GHz microwaves, on cognitive functions [15,33].

The immune system, which plays pivotal roles in defending against viruses and eliminating mutated cells, is sensitive to electromagnetic field radiation, such as microwave radiation [2,23]. However, the biological effects and their potential mechanisms are still controversial. Some studies explored the concept that microwaves could stimulate the proliferation of lymphocytes and promote the secretion of cytokines to enhance immune functions, while others reported inhibitory effects on immune functions due to the differences in power density, duration of exposure, and so on [12,13,34,35]. Our previous studies have reported that multifrequency microwaves of 1.5 GHz and 4.3 GHz decreased the white blood cells and lymphocytes in peripheral blood, suggesting immune suppression [36]. In this study, we also found that multifrequency microwaves of 1.5 GHz and 2.8 GHz injured structures of immune organs, which in turn decreased immune cells and reduced serum levels of cytokines and Igs in peripheral blood.

The interaction between microwaves and the biological system can produce both thermal and nonthermal effects. Microwave ablation mainly utilizes the thermal effects of microwaves. It has emerged as a minimally invasive and effective adjuvant approach for cancer therapy due to its safety and feasibility [37,38]. Microwave ablation has been widely used in the clinical treatment of hepatocellular carcinoma (HCC), renal cancer, osteoid osteoma, thoracic cancer, lung cancer, and others [39,40,41]. Moreover, microwave ablation has always been applied in the clinic together with immunotherapy [17,42]. Accumulating data suggest that microwave ablation can increase the infiltration of cytotoxic T lymphocytes (CTLs) into tumors, improve the secretion of Th1 cell cytokines, and decrease Th2 cytokines, suggesting that microwaves activate anti-tumor responses via multiple mechanisms [18,43]. Current data suggest that the thermal effects mainly contribute to the activation of anti-tumor immune responses after microwave ablation.

Until now, only a few groups have reported the nonthermal effects of microwaves on the immune system [34,44]. The accumulative effects of low-power communication microwaves with different frequencies have been analyzed in the immune system, but no significant alterations were detected [45,46,47]. Solid data about the accumulative effects induced by multifrequency microwaves are still lacking. Our group previously established stable animal models to evaluate the nonthermal effects of microwaves (Supplementary Figure S2), and the nonthermal effects on the hippocampus, heart, and testis were evaluated. Our data suggested that power-density-dependent injuries could be detected in these tissues [10,25,48,49]. In this study, we exposed rats to a multifrequency microwave of 1.5 GHz and 2.8 GHz, the body temperature was monitored both before and immediately after exposure, and no obvious changes were observed. And then, the nonthermal effects on the immune system were analyzed. Our data showed that 30 mW/cm^2^ multifrequency microwave exposure significantly decreased immune cells, including white blood cells, lymphocytes, monocytes, and neutrophils, in peripheral blood and decreased the levels of several cytokines. However, only slight changes could be detected in 5 mW/cm^2^ multifrequency microwave-exposed rats. Our data indicated that the multifrequency microwaves could induce immunosuppressive responses, which were positively associated with average power density.

Cytokines are critical effectors in regulating both anti-virus and anti-tumor immune responses via direct cytotoxicity and indirectly activating immune cells, such as B lymphocytes and natural killers [50,51]. We previously reported that multifrequency microwaves of 2.8 GHz and 9.3 GHz significantly increased the expression of IL-1α, IL-1β, and IL-4 at 6 h after exposure, which, in turn, up-regulated B lymphocytes at 7 d after exposure in peripheral blood [52]. However, no obvious changes of IL-1α, IL-1β, and IL-4 were observed at 6 h after exposure to the multifrequency microwave of 1.5 GHz and 2.8 GHz in this study. Moreover, obvious down-regulation of IL-1α and IL-1β, as well as TNF-α and IL-12p70, could be detected at 7 d after exposure, which might result in the decrease of lymphocytes at 7 d, 14 d, and 28 d after exposure.

Resonance is a pivotal event during microwave and biological systems. Resonance causes electrons or ions to vibrate the molecular bonds of biomacromolecules, such as proteins, which can impact the activity of proteins [6]. A polarized/coherent electromagnetic field can induce ion forced-oscillation and irregular gating of voltage-gated ion channels to disrupt intracellular ionic concentrations, resulting in DNA damage and ROS generation [53,54]. Inducing ROS has emerged as a potential mechanism underlying microwave-induced biological responses. Our previous studies and others have reported that microwaves can induce disorders of energy metabolism, companying decreased adenosine triphosphate (ATP) and increased ROS in neurons both in vitro and in vivo [55,56,57]. It has been reported that the induction of ROS after exposure to high-power microwave pulses can induce p53 activation and DNA damage in brain cells. MW pulsed signals from GSM900/UMTS test-mobile phone also elevate the ROS level in the hematopoietic stem or progenitor cells from umbilical cord blood (UCB); however, it did not cause DNA damage and cellular apoptosis [58,59]. The different responses might be attributed to the sensitivity of certain types of cells to ROS. Therefore, it is interesting to explore whether ROS contributes to microwave-induced injuries of the immune system in the future.

In conclusion, a multifrequency microwave dose of 1.5 GHz and 2.8 GHz injured both the structures and functions of the immune system, including the thymus, spleen, and mesenteric lymph nodes. Moreover, multifrequency microwave exposure also decreased the number of immune cells and reduced cytokines and Igs in peripheral blood. However, the mechanisms underlying the immune inhibitory responses to multifrequency microwaves still need to be uncovered in the future. Taken together, it will be necessary to develop an effective strategy to protect workers in special occupations who are exposed to low-level microwaves over the long term from microwave-induced nonthermal effects.

## Data Availability

Not applicable.

## Supplementary Materials

The following supporting information can be downloaded at: https://www.mdpi.com/article/10.3390/ijerph20064988/s1, Figure S1: The schematic of the experimental setup and exposure system; Figure S2: The rats’ anal temperature before and immediately after multi-frequency microwave of 1.5 GHz and 2.8 GHz exposure.
