# Supplementary material for: Accumulative Effects of Multifrequency Microwave Exposure with 1.5 GHz and 2.8 GHz on the Structures and Functions of the Immune System
Source: Int J Environ Res Public Health. 2023 Mar 12;20(6):4988. doi: 10.3390/ijerph20064988 (PMC10049199; doi:10.3390/ijerph20064988)
Supplement: Supplementary file 1 [file ijerph-20-04988-s001.zip › ijerph-2239318-supplementary.pdf]

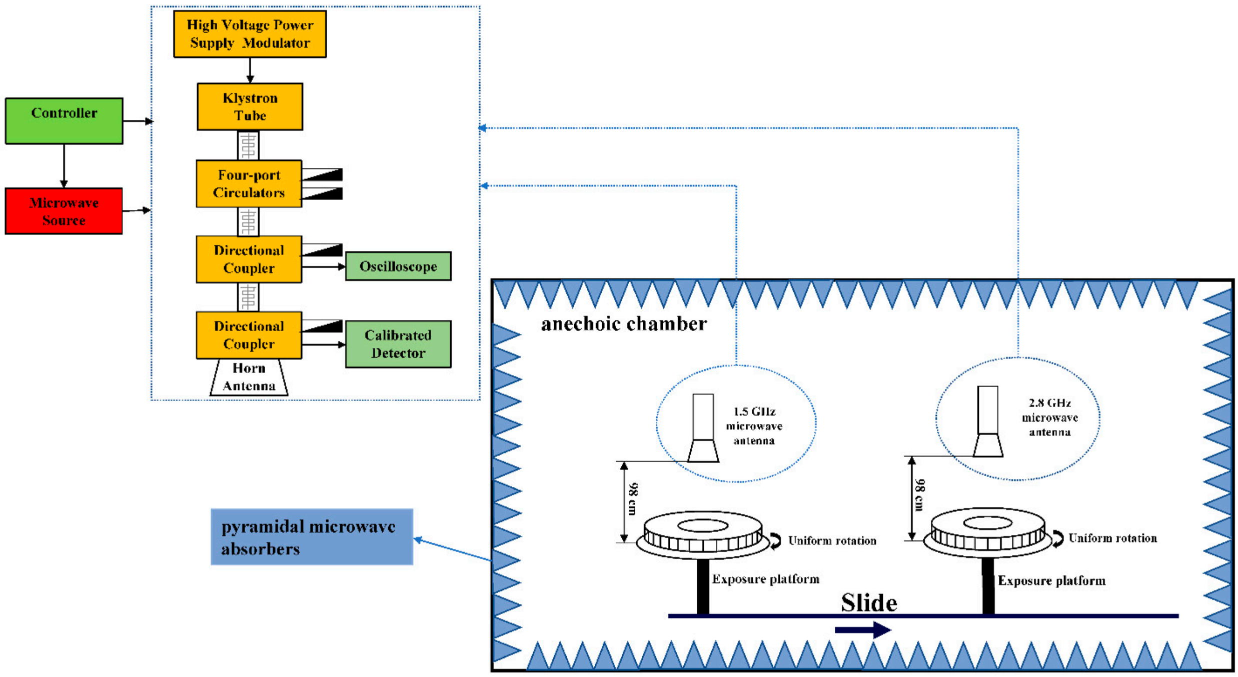

Supplementary Fig. S1. The schematic of the experimental setup and exposure system.

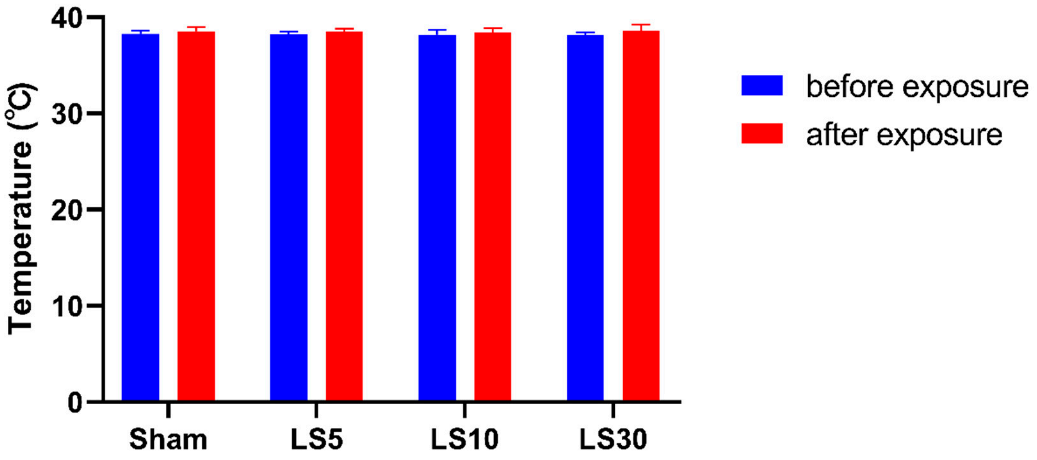

Supplementary Fig. S2. The rats' anal temperature before and immediately after multi-frequency microwave of 1.5 GHz and 2.8 GHz exposure. The anal temperatures of rats were measured before and immediately after microwave exposure by portable intelligent digital thermometer JM222 (Tianjin Jinming, China). Temperatures increased less than 1 °C after microwave exposure, and there was no significant difference. Data are shown as the mean±s.e.m.
